# Supplementary material for: Prefrontal tDCS is unable to modulate mind wandering propensity or underlying functional or effective brain connectivity
Source: Sci Rep. 2022 Oct 26;12:18021. doi: 10.1038/s41598-022-22893-8 (PMC9606118; doi:10.1038/s41598-022-22893-8)
Supplement: Supplementary file 1 — Supplementary Table S1. [file 41598_2022_22893_MOESM1_ESM.docx]

**Table S1.** Baseline group activity.

| **Contrast** | **Region (using AAL atlas)** | **x; y; z** | **k** | **z-score** | **F/t** |
| --- | --- | --- | --- | --- | --- |
| **Offline session 1 off-task > on-task** | Postcentral Gyrus | -41.5; -30.0; 54.0 | 571 | 4.377 | 6.089 |
|  | Extra-Nuclear | -29.0; -27.5; -3.5 | 27 | 3.945 | 5.151 |
|  | Outside BA | 1.0; -67.5; -46.0 | 138 | 3.899 | 5.060 |
|  | Outside BA | 1.0; -30.0; 1.5 | 60 | 3.894 | 5.051 |
|  | Cingulate Gyrus | -14.0; -27.5; 34.0 | 19 | 3.892 | 5.046 |
|  | Cuneus | 8.5; -87.5; 4.0 | 755 | 3.886 | 5.035 |
|  | Inferior Occipital Gyrus | -41.5; -90.0; -8.5 | 185 | 3.875 | 5.014 |
|  | Culmen | -29.0; -47.5; -28.5 | 14 | 3.651 | 4.590 |
|  | Thalamus | -16.5; -15.0; 4.0 | 90 | 3.626 | 4.544 |
|  | Outside BA | -29.0; -52.5; 69.0 | 67 | 3.589 | 4.476 |
|  | Fusiform Gyrus | 28.5; -87.5; -23.5 | 52 | 3.576 | 4.453 |
|  | Middle Temporal Gyrus | 51.0; -52.5; 6.5 | 71 | 3.479 | 4.282 |
|  | Cerebellar Tonsil | -41.5; -50.0; -48.5 | 38 | 3.366 | 4.090 |
|  | Cingulate Gyrus | -4.0; 22.5; 29.0 | 20 | 3.321 | 4.015 |
| **Offline session1 commission error > correct withhold** | Precentral Gyrus | -11.5; -25.0; 69.0 | 364 | 4.103 | 5.479 |
|  | Sub-Gyral | 31.0; -42.5; -3.5 | 308 | 4.067 | 5.403 |
|  | Precentral Gyrus | 26.0; -20.0; 61.5 | 79 | 3.876 | 5.015 |
|  | Insula | -44.0; -5.0; 14.0 | 35 | 3.873 | 5.008 |
|  | Medial Frontal Gyrus | 8.5; -15.0; 61.5 | 42 | 3.694 | 4.668 |
|  | Sub-Gyral | -34.0; -62.5; -1.0 | 82 | 3.649 | 4.585 |
|  | Precentral Gyrus | 11.0; -22.5; 74.0 | 33 | 3.624 | 4.540 |
|  | Middle Temporal Gyrus | 48.5; -75.0; 19.0 | 13 | 3.549 | 4.405 |
|  | Postcentral Gyrus | 28.5; -37.5; 51.5 | 18 | 3.488 | 4.298 |
|  | Sub-Gyral | 13.5; -30.0; 56.5 | 22 | 3.392 | 4.134 |
| **Interaction: online > offline x anodal > sham** | Sub-Gyral | -41.5; -5.0; -18.5 | 19 | 3.780 | 17.669 |
|  | Middle Occipital Gyrus | 38.5; -85.0; -1.0 | 44 | 3.505 | 15.154 |
|  | Superior Temporal Gyrus | 43.5; 2.5; -13.5 | 15 | 3.494 | 15.058 |
|  | Declive | 8.5; -70.0; -21.0 | 37 | 3.424 | 14.463 |
|  | Cuneus | -26.5; -87.5; 19.0 | 11 | 3.256 | 13.096 |
|  | Middle Occipital Gyrus | 31.0; -90.0; 11.5 | 13 | 3.252 | 13.061 |
|  | Sub-Gyral | -26.5; -75.0; 21.5 | 10 | 3.185 | 12.541 |
| **Effect of run commission error > correct withhold** | Insula | 46.0; 5.0; 6.5 | 28 | 3.596 | 15.863 |

*Results from the one sample t-test from the offline first session run of the SART at an uncorrected p<0.001 (peak level). Only clusters of 10 and above are displayed.*
